# The effects of use of medications on striatal dopamine transporter binding assessed by [^123^I]I-FP-CIT SPECT in patients with Parkinson’s disease from the PPMI cohort

**DOI:** 10.1007/s00259-026-08136-2

**Published:** 2026-08-15

**Authors:** Abdallah Nour Ali, Ilham Malik, Jan Booij, Youssef Chahid

**Affiliations:** 1 Department of Radiology and Nuclear Medicine, University of Amsterdam, Amsterdam UMC, Meibergdreef 9, Amsterdam, 1105 AZ The Netherlands; 2 Department of Pharmacy, University of Amsterdam, Amsterdam UMC, Amsterdam, The Netherlands

**Keywords:** Medication, Specific binding ratios, DaTSCAN, Dopamine transporter imaging, [^123^I]I-FP-CIT, PPMI

## Abstract

**Purpose:**

Several centrally acting medications may interfere with dopamine transporter (DAT) imaging using [¹²³I]I-FP-CIT SPECT, potentially leading to misinterpretation. We therefore evaluated the effects of several medications on striatal DAT binding assessed by [^123^I]I-FP-CIT SPECT in patients with Parkinson’s disease (PD).

**Methods:**

Data were obtained from the Parkinson’s Progression Markers Initiative (PPMI). PD patients with abnormal [¹²³I]I-FP-CIT SPECT scans, and available medication and clinical data, were included. The effects of bupropion, venlafaxine, methylphenidate, modafinil, and codeine were assessed. Striatal specific binding ratios (SBRs) of the caudate nucleus and putamen were analysed using multivariable linear regression models adjusted for relevant covariates.

**Results:**

Data from 752 PD patients were included. Use of the evaluated medications was associated with lower SBRs in the ipsilateral and contralateral caudate nucleus (-13.5% and -13.7%, respectively) and putamen (-6.5% and -8.2%, respectively; all *p* < 0.01). Medication-specific analyses showed the largest reductions for methylphenidate and modafinil, with relative reductions in caudate nucleus SBRs of up to -33.2% and -25.5%, respectively (both *p* < 0.01). In contrast, bupropion and venlafaxine were associated with smaller, but still significant, reductions, whereas no significant associations were observed for codeine.

**Conclusion:**

Use of methylphenidate and modafinil in particular is associated with significantly reduced striatal [¹²³I]I-FP-CIT binding in PD patients. Medication use should be carefully considered when interpreting DAT SPECT scans. Temporary discontinuation may be considered in selected cases when clinically appropriate.

## Introduction

Dopamine transporter (DAT) imaging using [^123^I]I-FP-CIT single-photon emission computed tomography (SPECT) is widely employed to assess nigrostriatal degeneration in Parkinson’s disease (PD) [1–6]. Specific striatal binding ratios (SBR) of [^123^I]I-FP-CIT SPECT provide an in-vivo proxy for presynaptic dopaminergic neuron integrity and are routinely used to support the diagnosis of PD, differential diagnosis and research stratification [7–10].

Several centrally acting drugs are known to interact with DAT binding [11–13]. Medications such as methylphenidate, modafinil and bupropion inhibit the DAT and may competitively reduce [^123^I]I-FP-CIT binding [12]. This pharmacological interference could lead to underestimation of striatal DAT availability as assessed in-vivo and potentially lead to false-positive results or exaggerated patterns of nigrostriatal dopaminergic deficit.

In a recent systematic review, we highlighted the potential for medication-related modulation of DAT imaging, and reported which medications may influence the visual read of [^123^I]I-FP-CIT SPECT scans [12]. However, the available clinical evidence remains limited and is largely based on small cohorts [12]. In the present study, we therefore evaluated the association between the use of medications, which may influence striatal DAT binding assessed by [^123^I]I-FP-CIT binding, in a large, well-characterized PD cohort from the Parkinson’s Progression Markers Initiative (PPMI) [14, 15]. We hypothesized that the use of medications, that were suggested to influence [^123^I]I-FP-CIT binding in our systematic review, is associated with reduced striatal DAT binding. This approach allows us to examine whether previously reported potential effects of medications on [^123^I]I-FP-CIT binding observed in small cohorts are reproducible in the large, well-characterized, PPMI dataset.

## Materials and methods

### Study design and population

We used data from the PPMI study, an international, multicentre observational study designed to identify biomarkers of PD progression [14, 15]. Although the PPMI cohort also includes healthy control participants, these individuals were not included in the present study. Patients were eligible if they had satisfied two inclusion criteria, namely; available [^123^I]I-FP-CIT SPECT SBR data and a documented medication use at the time of imaging. For each participant, only the first available DAT-SPECT scan with complete SBR data was included in the analyses to ensure independent observations.

The primary objective of the present study was to evaluate medications previously reported to substantially reduce striatal DAT binding [12, 16]. Cases were therefore selected if they were actively using any of the following drugs at the time of imaging: dexamphetamine, methamphetamine, ephedrine, dexmethylphenidate, methylphenidate, haloperidol, modafinil, armodafinil, phentermine, fentanyl, codeine, pseudoephedrine, bupropion or venlafaxine. For these patients the start and the stop dates of medication use were assessed to confirm active use at the time of [^123^I]I-FP-CIT SPECT imaging. The controls consisted of PD patients without any history of using these medications. Also, controls were selected to match for age, sex, disease duration, body mass index, motor severity score, anti-Parkinson medications use and selective serotonin reuptake inhibitor (SSRI) use. Controls were selected at a 3:1 ratio relative to the potential medication-use group.

### Outcomes measures

The primary outcome was striatal DAT binding, expressed as SBRs derived from [¹²³I]I-FP-CIT SPECT imaging, measured in the following regions-of-interest: caudate nucleus (ipsilateral and contralateral sides) and putamen (ipsilateral and contralateral).

### Statistical analysis

Continuous variables were compared between the two groups under study using independent-samples t-tests or Mann–Whitney U tests, as appropriate. Categorical variables were analysed using Pearson’s chi-square test. Variables with skewed distributions, including disease duration, putamen and caudate nucleus binding ratios, were log-transformed (log10; log(x + 1) for variables containing zero values) prior to parametric analysis to approximate normality. Associations between medication use and SBRs were assessed using general linear models. Covariates included age, sex, body mass index, disease duration of PD, and motor severity (MDS-UPDRS part III, Movement Disorders Society Unified Parkinson Disease Rating Scale), as these factors are known to influence striatal DAT availability [17–25]. In addition, variables reflecting comorbidity and medication use, including SSRI use [26] and anti-Parkinson medication use [12], were incorporated into the models.

Medication-specific analyses were restricted to patients receiving the investigated medication as monotherapy. Patients receiving more than one of the investigated medications were excluded from these analyses to avoid overlap between medication groups. Separate grouped analyses were performed to evaluate the effects of bupropion and/or venlafaxine and modafinil and/or methylphenidate. These grouped analyses included both patients receiving either medication as monotherapy and those receiving both medications concomitantly. This approach was chosen to increase the number of cases within these analyses and thereby improve statistical power.

Because SBR values were log10-transformed prior to the regression analyses, regression coefficients were converted to estimated relative percentage changes using the formula: percentage change = (10^β − 1) × 100. The resulting values represent the estimated relative change in SBR associated with medication use after adjustment for the covariates included in the model. To assess whether the medication-associated reduction in striatal [¹²³I]I-FP-CIT binding differed between the caudate nucleus and the putamen, an additional linear mixed-effects analysis was performed including region of interest (ROI), medication use, their interaction, and the same covariates as in the primary analyses.

Regression diagnostics were performed for the medication-specific subgroup analyses using Cook’s distance to assess the influence of individual observations on the regression estimates. A Cook’s distance > 1.0 was considered indicative of a potentially influential observation.

Non-parametric Mann–Whitney U tests were performed as a sensitivity analysis for medication subgroups with very small sample sizes (*n* < 10) to evaluate whether the observed differences in striatal SBR remained robust without relying on the assumptions of the linear regression models.

Statistical significance was defined as *p* < 0.05 (two-sided). Analyses were performed using IBM SPSS version 28.0.

## Results

### Study population

Data of a total of 752 PD patients were included, of whom 188 were classified as using potential drugs that may influence striatal [^123^I]I-FP-CIT binding (potential medication-use group) and 564 as non-users (controls). Only five potential drugs were finally included in the study since only these medications were used in the case group. These drugs are bupropion, venlafaxine, methylphenidate, modafinil and codeine. None of the PD patients were using other medications that potentially may influence striatal [^123^I]I-FP-CIT binding, such as dexamphetamine or haloperidol. Baseline characteristics were comparable between the two groups, except for a higher prevalence of anti-parkinsonian medication use in the control group (*p*-value < 0.01) (Table 1).

**Table 1:** Participants demographics and clinical characteristics

Characteristic	Potential medication-use group	Control group	*p*-value
Number of patients	188	564	
Age	62.1 ± 10.2	62.2 ± 9.5	0.89^*^
Sex (% male)	50.5%	50.5%	1.00^^^
Body mass index (kg/m^2^)	27.4 ± 4.7	27.0 ± 4.7	0.38^*^
Motor severity score (MDS-UPDRS-III)	18.1 ± 11.4	18.5 ± 8.1	0.66^*^
Disease duration of PD (month)	6.0 (2.5–19.0)^≠^	6.1 (3.0-14.1)^≠^	0.83^§^
Anti-Parkinson medication use	63.8%	74.5%	< 0.01^^^
SSRI use	35.1%	29.1%	0.12^^^
Included drugs:			
Bupropion	73	0	
Venlafaxine	61	0	
Methylphenidate	7	0	
Modafinil	9	0	
Codeine	6	0	
Bupropion and/or venlafaxine	147	0	
Modafinil and/or methylphenidate	31	0	

± = Mean ± SD, * = Independent-samples t-test, ^ = Pearson Chi-Square, §= Mann-Withney U test, ≠ = Median + interquartile range, PD = Parkinson’s disease; SSRI= selective serotonin reuptake inhibitor

### Overall effect of medication use

First, we evaluated the overall effect of the a priori selected medications on striatal [¹²³I]I-FP-CIT binding. Multivariable linear regression analyses showed that use of these medications was independently associated with significantly lower SBRs across all regions of interest (*p* < 0.01). The magnitude of the reduction was greater in the caudate nucleus than in the putamen. The relative reduction in SBR of -13.5% (95% CI -17.0% to -9.8%) and -13.7% (95% CI -17.0% to -10.1%) in both the ipsilateral and contralateral caudate nucleus respectively. In the contralateral putamen  -8.2% (95% CI -11.7% to -4.5%) and -6.5% (95% CI -9.8% to -2.9%) in the ipsilateral putamen (Table 2). The interaction between medication use and ROI was statistically significant (F(1, 2781) = 7.19, *p* = 0.01), indicating that the medication-associated reduction in striatal [¹²³I]I-FP-CIT binding was greater in the caudate nucleus than in the putamen.

**Table 2:** Overall effect of included drugs on the striatal binding ratios of [^123^I]I-FP-CIT

Region of interest	Relative change in SBR (%)	*P*-value^*^
Caudate nucleus		
ipsilateral	-13.5% (-17.0% to -9.8%)	< 0.01
contralateral	-13.7% (-17.0% to -10.1%)	< 0.01
Putamen		
ipsilateral	-6.5% (-9.8% to -2.9%)	< 0.01
contralateral	-8.2% (-11.7% to -4.5%)	< 0.01

Percentages are relative change in SBR (95% CI), adjusted for age, sex, body mass index, motor severity, PD disease duration, anti-parkinsonian medication use and SSRI use. Negative values indicate lower SBRs and positive values indicate higher SBRs relative to the control group

* = *P*-value for the unstandardized regression coefficient from multivariable linear regression analysis of log-transformed striatal binding ratios of [^123^I]I-FP-CIT

### Medication-specific effects

Secondly, we assessed the medication-specific effects of the five potential DAT-interacting drugs on the SBRs of [¹²³I]I-FP-CIT. Multivariable linear regression analyses demonstrated that methylphenidate and modafinil were associated with the largest reductions in SBRs after adjustment for covariates. Methylphenidate was associated with relative reductions in SBR ranging from -15.9% to -33.2% across the different regions of interest, with the largest reductions observed in the caudate nucleus. Similarly, modafinil was associated with relative reductions ranging from -14.9% to -25.5%, again with the strongest effects in the caudate nucleus (Table 3).

**Table 3:** Medication-specific effects, and effects of combined use of drugs, on the striatal binding ratios of [^123^I]I-FP-CIT

Medication	Caudate nucleus		Putamen	
	ipsilateral	contralateral	ipsilateral	contralateral
Bupropion (*n* = 73)	-13.9%*(-18.9% to -8.4%)	-14.3%*(-19.3% to -4.5%)	-4.5% (-9.6% to 1.2%)	-8.2%*(-13.5% to -2.3%)
Venlafaxine (*n* = 61)	-11.1%*(-16.4% to -5.6%)	-10.5%*(-15.7% to -4.9%)	-6.5%*(-11.5% to -1.1%)	-6.9%*(-12.3% to -0.9%)
Bupropion and/or venlafaxine (*n* = 147)	-12.1%*(-15.9% to -8.2%)	-12.1%*(-15.9% to -8.2%)	-5.2%*(-9.0% to -1.4%)	-7.1%*(-11.1% to -2.9%)
Methylphenidate (*n* = 7)	-33.2%* (-43.9% to -20.4%)	-31.5%* (-42.3% to -18.5%)	-15.9%* (-28.4% to -1.1%)	-18.2%* (-31.3% to -2.5%)
Modafinil (*n* = 9)	-25.5%* (-35.0% to -14.7%)	-24.5%* (-34.1% to -13.7%)	-14.9%* (-25.0% to -3.4%)	-18.9%* (-29.4% to -7.1%)
Modafinil and/or methylphenidate (*n* = 31)	-24.7%* (-30.5% to -18.2%)	-24.1%* (-30.2% to -17.8%)	-14.1%* (-20.4% to -7.5%)	-16.2%* (-22.7% to -9.2%)
Codeine (*n* = 6)	19.1% (-1.6% to 44.5%)	14.8% (-5.2% to 39.0%)	12.2% (-6.2% to 34.3%)	15.6% (-4.7% to 40.6%)

Percentages are relative change in SBR (95% CI), adjusted for age, sex, body mass index, motor severity, PD disease duration, anti-parkinsonian medication use and SSRI use. Negative values indicate lower SBRs and positive values indicate higher SBRs relative to the control group

* = *P*-value < 0.05 for the unstandardized regression coefficient from multivariable linear regression analysis of log-transformed striatal binding ratios of [^123^I]I-FP-CIT

Bupropion and venlafaxine showed more moderate, but still significant, reductions in SBR, corresponding to relative decreases of approximately -4.5% to -14.3% across the different regions of interest (Table 3). No statistically significant associations were observed for codeine, despite estimated relative increases in SBR ranging from 12.2% to 19.1%, as none of the regression coefficients reached statistical significance (Table 3).

Cook’s distance values ranged from 0.02 to 0.46 across all medication-specific models and regions of interest, with none exceeding the predefined threshold of 1.0. In the non-parametric sensitivity analyses (Mann–Whitney U tests), significant reductions in striatal SBR were observed across all regions for methylphenidate and modafinil (all *p* < 0.05). For codeine, significantly higher SBR values were observed in the ipsilateral caudate nucleus and ipsilateral putamen (both *p* < 0.05), whereas no significant differences were observed in the contralateral caudate nucleus or contralateral putamen.

Finally, we evaluated the effect of combined drug use. The combinations consisted of the concurrent use of one or two of the study drugs that may potentially influence striatal [¹²³I]I-FP-CIT binding. Combined drug use was also associated with significantly reduced SBRs in both the caudate nucleus and putamen. The combination of methylphenidate and/or modafinil was associated with the largest reductions, corresponding to relative decreases ranging from -14.1% to -24.7% across the different regions of interest. In contrast, the combination of bupropion and/or venlafaxine was associated with more moderate reductions, corresponding to relative decreases ranging from  -5.2% to -12.1% (Table 3).

## Discussion

In this study, we confirmed in a large cohort from the PPMI study that use of medications such as bupropion, venlafaxine, methylphenidate and modafinil may significantly decrease the SBRs of [^123^I]I-FP-CIT both in the caudate nucleus and putamen. These findings may have important clinical implications, as they underscore the need to account for the use of these medications when interpreting [^123^I]I-FP-CIT SPECT scans. In line with our previous recommendations, temporary discontinuation of these agents prior to DAT imaging should be considered to minimize pharmacological interference and reduce the risk of misinterpretation [12].

Several of the drugs examined in the present study are known to interact, directly or indirectly, with the DAT [12, 13]. Methylphenidate exhibits relatively high affinity for the DAT (Ki ≈ 40–100 nM) [27, 30] and increase extracellular dopamine primarily through competitive DAT blockade [28–31], whereas modafinil shows lower, micromolar affinity (Ki ≈ 2–6 µM) [32, 33], but still achieves measurable DAT occupancy at clinically relevant doses [32–34, 35–37]. Bupropion has low-to-moderate affinity for the DAT (Ki ≈ 500–2,000 nM) and has been reported to reduce striatal [¹²³I]FP-CIT binding in vivo [35–39]. Acute or subacute DAT occupancy by these agents may reduce radiotracer binding and thereby mimic dopaminergic deficits [33–39].

In contrast, venlafaxine displays negligible affinity for the DAT (Ki > 10 µM), but may still influence DAT-SPECT measurements indirectly [40, 41]. As a serotonin–norepinephrine reuptake inhibitor, venlafaxine modulates monoaminergic neurotransmission and may thereby indirectly affect striatal DAT availability, although mechanistically this interaction is not well understood [16, 42].

In the present study we showed that bupropion, venlafaxine, methylphenidate and modafinil decrease the striatal [¹²³I]I-FP-CIT binding both in the caudate nucleus and putamen in PD patients. Importantly, the observed effects were highly consistent across both the ipsilateral and contralateral caudate nucleus and putamen. This bilateral pattern supports the biological plausibility of a systemic pharmacological effect on DAT binding, as these drugs would not be expected to selectively affect DAT binding in only one hemisphere. This uniform striatal reduction of binding fits well with the finding of a previous study by Apostolova and co-workers on the effects of bupropion and venlafaxine on striatal [¹²³I]I-FP-CIT binding [16]. Interestingly, the use of methylphenidate and modafinil have a two-fold greater effect in reducing the SBRs compared to bupropion and venlafaxine. This is to be expected from the interaction mechanism of both drugs with the DAT as described above. The magnitude of reduction was greater in the caudate nucleus than in the putamen, suggesting regional differences in sensitivity to detect pharmacological DAT blockade in PD patients. Not even a significant effect was observed on ipsilateral putamen binding by bupropion. This is maybe due to the pronounced loss of [^123^I]I-FP-CIT binding in the putamen in PD, which may induce a so-called floor effect.

In the present study, the use of codeine was not associated with significantly decreased striatal [¹²³I]I-FP-CIT binding. This finding differs from previous work by *Hou et al.*, in which reduced striatal DAT availability was observed in a [^99m^Tc]TRODAT SPECT study in addicted patients to codeine-containing cough syrup as compared to age-matched controls [43]. However, an important distinction is that the prior study involved individuals with opioid dependence, a condition that itself may alter dopaminergic neurotransmission, including lower striatal DAT binding [44]. Therefore, the observed reductions in striatal DAT binding in that context may reflect neurobiological effects of drug addiction rather than a direct pharmacological effect of codeine. In our present cohort, codeine use likely reflects therapeutic (in combination with acetaminophen), low-dose exposure, which may not significantly interact with DAT binding. Additionally, codeine primarily acts on µ-opioid receptors and has no strong known affinity for the DAT, supporting the absence of a measurable effect on DAT SPECT binding in our study. Consistent with the primary analysis, the non-parametric sensitivity analysis using the Mann–Whitney U test provided no evidence of a substantial reduction in striatal [¹²³I]I-FP-CIT binding associated with codeine use. Although these findings should be interpreted cautiously given the small number of codeine users, they suggest that therapeutic codeine use is unlikely to substantially reduce striatal [¹²³I]I-FP-CIT binding and thereby cause a false-positive DAT-SPECT interpretation.

A major strength of this study is the use of a large, well-characterized PD cohort derived from the PPMI, providing sufficient statistical power to detect modest associations between medication use and [¹²³I]I-FP-CIT binding. The availability of detailed clinical and demographic data enabled adjustment for multiple relevant confounders, including age, sex, disease duration of PD, motor severity and other medication use such as SSRIs. In addition, the medication-specific analyses offer clinically meaningful insights into differential pharmacological effects on DAT binding, thereby enhancing the translational relevance of our findings for both clinical practice and research settings.

Several limitations should be acknowledged. First, detailed information regarding medication dosage, frequency, and duration of use was not always available in the PPMI database, limiting the ability to assess potential dose-response relationships. Second, the possibility of confounding by indication should be acknowledged. Psychiatric comorbidities and the underlying conditions for which the investigated medications were prescribed were not included as covariates in our multivariate linear regression model. Therefore, residual confounding cannot be excluded. Conditions such as major depressive disorder, ADHD, narcolepsy, or fatigue-related symptoms may themselves influence dopaminergic neurotransmission and DAT availability, which could affect striatal SBR measurements. Consequently, the observed reduction in striatal SBR among medication users may not solely reflect pharmacological effects of the medications but could also partly relate to the underlying indication for treatment [45]. Third, longitudinal analyses of participants who initiated or discontinued the investigated medications between serial DAT-SPECT examinations, as well as analyses in healthy controls, could not be performed due to the limited number or absence of eligible participants. Finally, some drug-specific subgroups were relatively small. Although statistically significant reductions in DAT binding were observed in some of these subgroups, the findings should be interpreted with caution given the limited sample sizes. Furthermore, the absence of statistically significant associations in other subgroups should not be interpreted as evidence of no effect, as the small sample sizes may have limited the statistical power to detect an association (i.e., type II errors cannot be excluded). Therefore, the findings for the smaller medication subgroups require confirmation in larger studies.

## Conclusion

The centrally acting drugs methylphenidate, modafinil, bupropion, and venlafaxine are independently associated with reduced striatal [¹²³I]I-FP-CIT binding in patients with Parkinson’s disease. These findings highlight the importance of considering concomitant medication use when interpreting DAT-SPECT scans. Temporary discontinuation of these medications may be considered on an individual basis when clinically appropriate, taking into account the potential benefits and risks. However, particularly if discontinuation of these medications appears to be problematic, one has to take into account that these medications may cause a uniform reduction of striatal [¹²³I]I-FP-CIT binding, which can often be clearly distinguished from the typical findings in Parkinson’s disease (i.e., asymmetric striatal binding and lower binding in the putamen than in the caudate nucleus).

## Data Availability

The datasets generated during and/or analysed during the current study are available from the corresponding author on reasonable request.
